# NADPH Oxidase-Dependent Production of Reactive Oxygen Species Induces Endoplasmatic Reticulum Stress in Neutrophil-Like HL60 Cells

**DOI:** 10.1371/journal.pone.0116410

**Published:** 2015-02-10

**Authors:** Wilson Mitsuo Tatagiba Kuwabara, Liling Zhang, Irmgard Schuiki, Rui Curi, Allen Volchuk, Tatiana Carolina Alba-Loureiro

**Affiliations:** 1 Department of Physiology and Biophysics, Institute of Biomedical Sciences, University of São Paulo, São Paulo, Brazil; 2 Department of Physiology, University of Toronto, Toronto, Ontario, Canada; The Hospital for Sick Children and The University of Toronto, CANADA

## Abstract

Reactive oxygen species (ROS) primarily produced via NADPH oxidase play an important role for killing microorganisms in neutrophils. In this study we examined if ROS production in Human promyelocytic leukemia cells (HL60) differentiated into neutrophil-like cells (dHL60) induces ER stress and activates the unfolded protein response (UPR). To cause ROS production cells were treated with PMA or by chronic hyperglycemia. Chronic hyperglycemia failed to induce ROS production and did not cause activation of the UPR in dHL60 cells. PMA, a pharmacologic NADPH oxidase activator, induced ER stress in dHL60 cells as monitored by IRE-1 and PERK pathway activation, and this was independent of calcium signaling. The NADPH oxidase inhibitor, DPI, abolished both ROS production and UPR activation. These results show that ROS produced by NADPH oxidase induces ER stress and suggests a close association between the redox state of the cell and the activation of the UPR in neutrophil-like HL60 cells.

## Introduction

Neutrophils are essential components of the innate immune system and have an important role in initiating and sustaining the inflammatory process. These cells synthesize proteins that participate in their own effector functions and in the inflammatory response, such as polypeptides, cytokines, chemokines, growth factors and interferons [1]. Neutrophils depend on the activation of NADPH oxidase [2] and hence the generation of reactive oxygen species (ROS) for their microbicidal activity [3; 4]. The ingestion of dead neutrophils by macrophages is the main mechanism to remove neutrophils recruited to the inflamed site and, thus, to promote the resolution of inflammation [5]. The high demand for the production of proteins and inflammatory responses requires the endoplasmatic reticulum (ER), an important organelle to maintain cell homeostasis [6].

The ER is present in all eukaryotic cells and is responsible for secretory and membrane protein biosynthesis. The lumen of the ER has a unique microenvironment and various protein folding chaperones that promote secretory protein biosynthesis and folding. The ER is the primary intracellular calcium reservoir and has a more oxidizing environment relative to the cytosol. High levels of intraluminal calcium are required for proper function of various chaperone proteins [7] and an oxidizing environment is required for efficient disulfide bond formation. Alterations in the ER microenvironment can result in ER stress caused by the accumulation of unfolded proteins. Eukaryotic cells respond to ER stress by activation of signaling cascades known as the Unfolded Protein Response (UPR). The UPR is detailed in some recent reviews [8–11].

Briefly, the ER stress response involves activation of three ER components: Inositol-Requiring kinase 1 (IRE1), double-stranded RNA-activated protein kinase-like ER kinase (PERK) and Activating transcription factor 6 (ATF6) [7; 12; 13]. When the concentration of unfolded proteins increases in the lumen of the ER, the chaperone Glucose Regulated Protein 78 (GRP78) (also named BiP) dissociates from the luminal domains of PERK, IRE1 and ATF6 to bind to unfolded proteins and promote protein folding. This causes activation of UPR pathways as follows: IRE1 oligomerizes, leading to autophosphorylation of its cytoplasmic domain and activation of the IRE1 endoribonuclease domain [10]. This results in cleavage of the X-box binding protein (XBP1) mRNA to remove a 26 nucleotides intron. The mRNA is re-ligated generating spliced XBP1 mRNA (sXBP1), which is efficiently translated. XBP1 is a transcription factor that activates many genes such as chaperones, ER associated degradation components and secretory pathway genes. PERK pathway activation involves oligomerization and autophosphorylation, leading to activation of the PERK kinase domain that phosphorylates Ser51 of the subunit of eukaryotic translation initiation factor 2 (eIF2α) [7]. Although the phosphorylation of eIF2α inhibits general protein synthesis, translation of select mRNAs including Activating Transcription Factor 4 (ATF4) is increased [12]. ATF4 belongs to the cAMP-response element binding (CREB) family of transcription factors and activates genes involved in oxidative stress suppression, metabolism and transport of amino acids. ATF6 activation involves translocation to the Golgi apparatus, where it is cleaved by Site-1 (S1P) and Site-2 (S2P) proteases that release a soluble 50-kDa domain (ATF6p50) protein. ATF6p50 migrates to the nucleus and activates the transcription of many genes involved in ER quality control, including GRP78 and GRP94 [10; 13].

ROS can activate UPR by changing the redox state in the ER lumen. ROS are also produced by the ER during basal cell metabolism and are increased during ER stress [14; 15]. Several cell types and particularly phagocytes such as neutrophils, express proteins of the Nox family and produce ROS by using NADPH [15–17]. The NADPH oxidase is an enzyme complex consisting of cytoplasmic proteins (p40^phox^, p47^phox^ and p67^phox^) and membrane proteins (gp91^phox^ or Nox2 and p22^phox^) to form a flavo-hemoprotein known as cytochrome b558 [18; 19]. NADPH oxidase transfers an electron of the complex to the oxygen molecule in the phagosome or in the cytosol, generating superoxide anion [20–23] and hydrogen peroxide, which is formed by spontaneous dismutation or by superoxide dismutase (SOD) activity [3; 24]. Most of the generated hydrogen peroxide is consumed by neutrophil myeloperoxidase [25; 26]. This enzyme catalyzes the formation of HOCl by oxidation of chloride ions [27; 28], the primary oxidant bactericidal agent produced by neutrophils [23; 29]. Some studies have shown that ROS produced by the NADPH oxidase are important mediators in the activation of ER stress [14; 15; 30–32]. However, the contribution of NADPH oxidase in causing ER stress in neutrophils has not been studied. Here we found that activation of NADPH oxidase by Phorbol-12-Myristate-13-Acetate (PMA) and the consequent increase in ROS production induced ER stress and activation of the UPR in neutrophil-like cells.

## Materials and Methods

### HL60 cell culture and differentiation

Human HL-60 cells were obtained from American Type Culture Collection (ATCC) (Manassas, VA, USA) and grown in endotoxin-free RPMI 1640 medium containing 5.5 mM glucose and 10% heat-inactivated FBS at 37°C in a 5% CO_2_ atmosphere. Media was changed every 3 days. To differentiate HL60 cells into neutrophil-like cells (dHL60), 1.25% DMSO was added to the media for 6 days as previously reported [3, 4]. After the differentiation, dHL-60 cells (1x10^6^ cells/mL) were cultured for 24 h in 5.5 mM [normal glucose (NG)] or 25 mM glucose [high glucose (HG)]. The nonphysiological sugar, 19.5 mM mannitol (MN), was used to examine the impact of the osmotic pressure exerted by glucose. Cell line passage numbers used were between 11 and 28.

### Flow cytometric analysis

To assess the production of ROS, dHL60cells were labeled with 10μM DHR 123 (Dihydrorhodamine 123) and stimulated with 200 nM PMA or PMA+ DPI (10μM) (Diphenyleneiodonium). For flow cytometric quantification of ROS, cells (1.5 x 10^6^) were incubated with or without DPI for 20 min stimulation with PMA. PMA was added 15 min before the analysis. Undifferentiated HL60 cells were used as a negative control. Following treatments, flow cytometric analyses were conducted on a FACSLSRII (Becton Dickinson, San Jose, CA, USA) flow cytometer. Cells were excited at 488 nm and emitted light measured at 510/20 nm. Data analysis was performed using the BD FACS Diva software version 6.0.

### Intracellular calcium concentration

dHL60 cells (1.5 x 10^6^) were incubated for 24h under the following conditions: NG, HG and MN. Then, cells were labeled with Indo-1-AM (5μM) for one hour at 37°C. After incubation, cells were washed with Ca^2+^ buffer (150mM NaCl, 4mM KCl, 25mM HEPES, 3mM CaCl_2_, 5mM pyruvate, 10mM glucose, pH7.3) and kept at room temperature prior to analysis. For calcium influx measurements, dHL60 were resuspended in Ca^2+^ buffer with 1mg/mL albumin (BSA). For ER calcium content, cells were washed and kept in Ca^2+^ free buffer (150mM NaCl, 4mM KCl, 25mM HEPES, 5mM pyruvate, 10mM glucose, 2mM EGTA, pH7.3). After labeling the cells with Indo-1-AM, intracellular calcium changes were monitored by fluorimetry (F-2500, HITACHI) at 37°C under constant agitation. PMA (1μM) or fMLP (Formyl-Methionyl-Leucyl-Phenylalanine) (1μM) were added to stimulate calcium influx. The ionophore, ionomycin (1μM) and magnesium chloride (2mM) (MnCl_2_) were used in the ER calcium content measurements and to determine the maximum and the minimum concentration of intracellular calcium, respectively. Intracellular calcium was calculated as previously reported [33].

### XBP-1 mRNA splicing

Total RNA was isolated from dHL60 cells (1,5 x 10^6^) using TRIzol reagent (Invitrogen) and RNeasy Mini Kit (Qiagen). The RNA was reverse transcribed to single-stranded cDNA using the High-Capacity cDNA reverse transcription kit (Applied Biosystems). The resulting cDNA was used for the PCR analysis. Human XBP-1 cDNA was amplified by OneStep RT-PCR kit (Qiagen) using primers that flank the intron excised by IRE1 exonuclease activity as previously described (21). Primer sequences used to amplify human XBP-1 were: 5´-TTA CGA GAG AAA ACT CAT GGC C-3´and 5´- GGG TCC AAG TTG TCC AGA ATG C -3´. The protocol used for the RT-PCR was as follows: 50°C (30 min); 95°C (15 min); 35 cycles of 94°C (1 min), 55°C (1 min), 72°C (1min); 72°C (10 min). RT-PCR products were resolved on a 3% agarose gel and visualized using ethidium bromide.

### Western blot analysis

Cells (1 x 10^7^) were transferred to tubes and centrifuged at 1,200 rpm for 10 min at 4°C. The pellet was washed with 1 ml of cold phosphate-buffered saline (PBS) and centrifuged at 1,200 rpm for 10 min at 4°C. The pellet was resuspended in 60μl of Triton X100 lysis buffer. Proteins were resolved by SDS-PAGE and transferred to nitrocellulose membranes. The membranes were blocked for 1h at room temperature with 5% skim milk and incubated with the specific primary antibodies overnight. Following incubation with secondary antibody conjugated to horseradish peroxidase, the bands were detected with the enhanced chemiluminescence system (Amersham Biosciences). Immunoblots were scanned and quantified using ImageJ software. ATF4 goat polyclonal antibody (ab1371) was purchased from Abcam. Bip rabbit polyclonal (3183) and phospho-eIF2α rabbit polyclonal (9721) antibodies were purchased from Cell Signaling. GADD34 rabbit polyclonal antibody (sc-8327) was purchased from Santa Cruz Biotechnology, and the γ-tubulin monoclonal antibody (T6557) was purchased from Sigma Aldrich.

### Real time polymerase chain reaction

Total RNA was obtained from 1x10^7^ dHL60 by the guanidine isothiocyanate extraction method [34], using TRIzol Reagent (Invitrogen, Carlsbad, CA, USA) followed by isolation using RNeasy mini kit (Qiagen). The purity was assessed by the 260/280 nm ratio and the quantity measured at 260 nm. The cDNA was synthesized from total RNA (1.0 μg) using the High capacity kit (Invitrogen). The following primers were used: ATF4 5´-GACCGAAATGAGCTTCCTGA-3´ and 5´-ACCCATGAGGTTTGAAGTGC-3´; GRP78 5´-GCCTGTATTTCTAGACCTGCC-3´ and 5´-TTCATCTTGCCAGCCAGTTG-3´; C/EBP homologous protein (CHOP) 5´-CTGCTTCTCTGGCTTGGCTG-3´ and 5´-GCTCTGGGAGGTGCTTGTGA-3´; ER-localized DnaJ 4 (ERdJ4) 5´-CGCCAAATCAAGAAGGCCT-3´ and 5´-CAGCATCCGGGCTCTTATTTT-3´; Growth Arrest DNA damage protein 34 (GADD34) 5´-GGAGGAAGAGAATCAAGCCA-3´ and 5´-TGGGGTCGGAGCCTGAAGAT-3´; Glyceraldehyde-3-phosphate dehydrogenase GAPDH 5´-CCACCCATGGCAAATTCCATGGCA-3´ and 5´-TCTAGACGGCAGGTCAGGTCCACC-3´; Homocysteine-induced ER protein (Herp) 5´-CACCGCGACTTGGAGCTGAGTGG-3´ and 5´-TCTGTGGATTCAGCCACCTTGG-3´; 18S 5´-GCAATTATTCCCCATGAACG-3´ and 5´-GGGACTTAATCAACGCAAGC-3´. Real-time PCR analysis was performed using the SyBR Green JumpStart kit (Sigma Aldrich) in a Rotor Gene 6000 equipment (Corbett Research, Mortlake, Australia). Gene expression was performed by 2^-ΔΔCT^ [35,36], using GAPDH and 18S genes as inner controls.

### Data Analysis

Results are presented as means ± S.E.M. Statistical significance was assessed by two way ANOVA followed by the Bonferroni test. p ≤ 0.05 was considered statistically significant.

## Results

### PMA activates NADPH oxidase and increases ROS production

HL60 cell is a tumor cell line that was isolated from a single patient with acute promyelocytic leukemia and can be differentiated *in vitro* into a variety of blood cell types [37]. To differentiate them into neutrophils polar components such as DMSO or retinoic acid are used. After differentiation, although they are not identical to primary neutrophils, neutrophil-like HL60 cells (dHL60) express all the components of NADPH oxidase and its functionality is similar to primary neutrophils [37–40].

**Fig 1:**
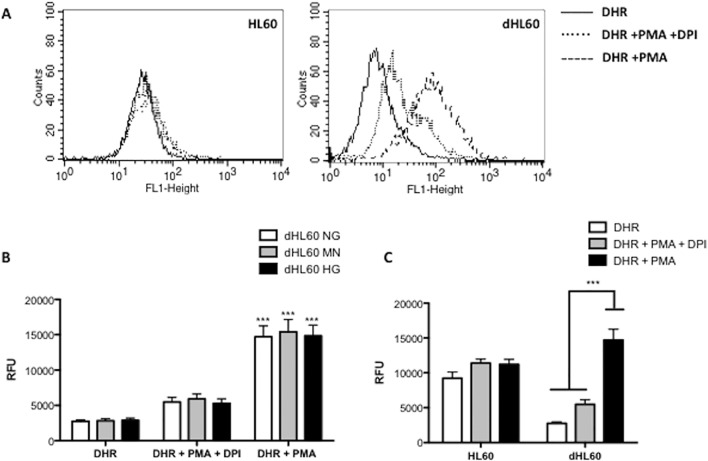
Effect of hyperglycemia and PMA on ROS production in neutrophil-like HL60 cells (dHL60) and non-differentiated HL60 cells. PMA (1μM) triggered the production of ROS only in dHL60 (A; C). Hyperglycemia did not affect the ROS production in neutrophil-like HL60 cells (B). DPI (10 μm) was used as NADPH oxidase inhibitor. [NG] normoglycemic media (5.5 mM of glucose); [MN] Mannitol enriched media (5.5 mM of glucose + 19.5 mM of mannitol); [HG] Hyperglycemic media (25 mM of glucose). DHR (10 μM) was used to monitor ROS production by flow cytometry. Graphs show median of fluorescence ± S.E.M. Results are from 6 independent experiments. (***) Indicate p <0.001.

dHL60 were used as a model to study the effect of ROS production on ER stress and UPR activation. Undifferentiated HL60 cells were used as a negative control; since they express nox proteins at a lower level than in dHL60 [41] and ROS production is not expected to be increased by PMA. Compared to dHL60, undifferentiated HL60 cells have higher basal ROS levels, which likely reflects a higher metabolic rate [15, 42] (Fig. 1A). Although chronic hyperglycemia has been shown to increase ROS levels in various cell types [43–46] it failed to increase ROS production in dHL60 cells as detected by DHR 123 [47] (Fig. 1B). However, we observed a 5-fold increase in ROS production after PMA in dHL60 cells but not in undifferentiated HL60 cells (Fig. 1C). ROS production stimulated by PMA in dHL60 cells was mostly NADPH oxidase-dependent since DPI, a specific inhibitor of the NADPH oxidase [16], significantly inhibited ROS production (Fig. 1A,C).

### PMA does not affect cellular calcium levels

**Fig 2:**
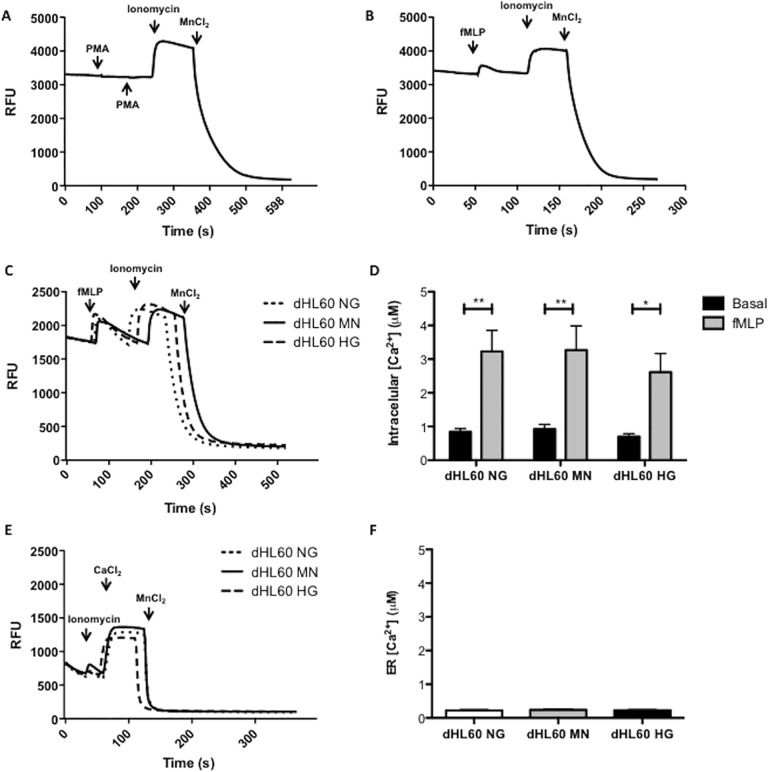
Effect of PMA, fMLP and hyperglycemia on calcium influx and ER calcium content in neutrophil-like HL60 cells (dHL60). PMA (1 μM) (A) did not alter calcium dynamics in dHL60 cells. fMLP (1 μM) (B-D) promoted calcium influx in neutrophil-like HL60 cells; and hyperglycemia (C) did not disturb the calcium intake by fMLP stimulation. ER calcium content in dHL60 was not altered by hyperglycemia (E; F). Calcium dynamics were monitored by Indo-1-AM (1 μM) using fluorometry. [NG] normoglycemic media (5.5 mM of glucose); [MN] Mannitol enriched media (5.5 mM of glucose + 19.5 mM of mannitol); [HG] Hyperglycemic media (25 mM of glucose). Graphs show fluorescence intensity during the time of analysis (A; B; C; E). Histograms show the mean intracellular calcium concentration ± S.E.M. (D) and the mean ± S.E.M. of ER calcium content (F). Results are from 4 independent experiments. (**) Indicate p <0.01 and (*) indicate p<0,05.

PMA activates PKC, which promotes the assembly of NADPH oxidase proteins [48]. ER stress can be caused by depletion of ER calcium stores as can be pharmacologically-induced using thapsigargin [46]. To examine if PMA or high glucose affect cellular calcium levels, intracellular calcium dynamics were monitored by using Indo-1-AM an ester of the calcium sensitive dye Indo-1 [47]. PMA failed to cause calcium influx (Fig. 2A) compared to fMLP used as a positive control (Fig. 2B). Similarly, chronic hyperglycemia did not cause variations in ER calcium content nor differences in calcium influx when cells were stimulated with fMLP (Fig. 2C-F).

### PMA induces ER stress by activating the NADPH oxidase

**Fig 3:**
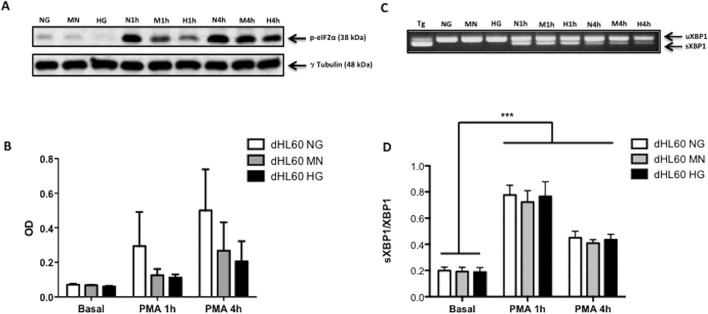
Effect of hyperglycemia and PMA on eIF2α phosphorylation and splicing of XBP1 mRNA in neutrophil-like HL60 cells (dHL60). Hyperglycemia failed to modulate eIF2α phosphorylation (A; B) and splicing of XBP1 (C; D). Splicing of XBP1 mRNA was caused by PMA (1 μM) stimulus (1 and 4h) (C; D). [NG] normoglycemic media (5.5 mM of glucose); [MN] Mannitol enriched media (5.5 mM of glucose + 19.5 mM of mannitol); [HG] Hyperglycemic media (25 mM of glucose). Histogram (B) shows the mean ± S.E.M. optical density (OD) of the protein bands (A). γ Tubulin was used as loading control. (C) Complementary cDNA bands of unspliced XBP1 (uXBP1) (top band) and spliced (sXBP1) (bottom band). Results is representative of 3 independent experiments. [Tg] Positive control (1 μM Thapsigargin, 1h); [N1h] NG + 1h PMA; [M1h] MN + 1h PMA; [H1h] HG + 1h PMA; [N4h] NG + 4h PMA; [M4h] MN + 4h PMA; [H4h] HG + 4h PMA.

To examine if PMA or high glucose caused ER stress we monitored activation of the PERK and IRE1 UPR pathways. Levels of phospho-eIF2α and spliced XBP1 mRNA were measured in dHL60 incubated in high glucose alone or treated with PMA for 1 and 4 h in normal or high glucose. Hyperglycemia alone failed to cause UPR activation (Fig. 3A-D). However, PMA caused an increase in spliced XBP1 mRNA levels in all conditions (normal glucose, mannitol control and high glucose) (Fig. 3C-D).

**Fig 4:**
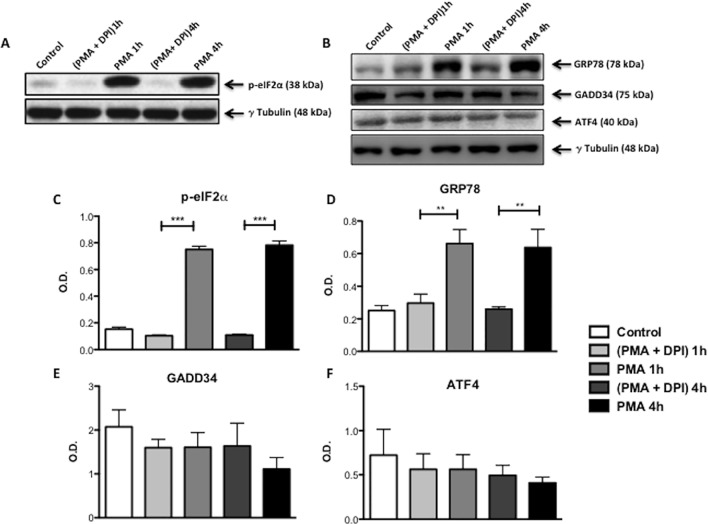
Effect of ROS production on UPR regulated proteins in neutrophil-like HL60 cells (dHL60). **** PMA (1 μM) treatment for 1 and 4h increased phosphorylated eIF2α (A; C) and GRP78 protein levels (B; D). This was blocked by DPI (10 μM), a NADPH oxidase inhibitor (A; B; C; D). GADD34 and ATF4 protein content was not altered by PMA (E; F). Histograms (C; D; E; F) show the mean ± S.E.M. of the optical density (OD) of the protein bands. γ Tubulin was used as loading control. Results are from at least 3 independent experiments. (***) Indicates p <0.001 and (**) Indicates p <0.01.

**Fig 5:**
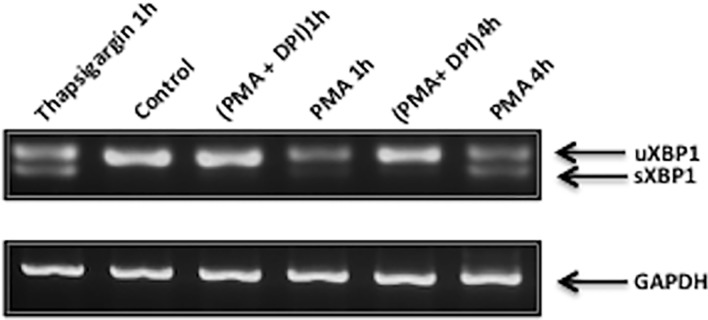
Effect of ROS production on the IRE1α/XBP1 pathway in neutrophil-like HL60 cells (dHL60). PMA (1 μM) treatment for 1h and 4h caused an increase in spliced XBP1 (sXBP1) mRNA content as monitored by RT-PCR (top panel). DPI (10 μM) inhibited the splicing of XBP1 mRNA. GAPDH was used as a loading control (bottom panel). Results are from 3 independent experiments. Thapsigargin (1μM, 1h) was used as a positive control for ER stress-induced XBP1 splicing.

Next we examined whether the activation of PERK and IRE1 by PMA in dHL60 cells was due to ROS produced by NADPH oxidase activation. PMA caused an increase in phospho-eIF2α levels after 1 and 4 hours of treatment and DPI abolished this effect (Fig. 4A,C). However, the levels of the PERK pathway regulated proteins GADD34 and ATF4 were not changed with PMA treatment (Fig. 4B,E,F). The UPR regulated ER chaperone GRP78 was also investigated. Treatment with PMA for 1 and 4 h increased the protein levels of GRP78 and this was completely prevented by the NADPH oxidase inhibitor DPI (Fig. 4B,D). To investigate the IRE1 pathway, levels of sXBP1 mRNA were analyzed. PMA increased the levels of sXBP-1 after 1 and 4h of treatment and this effect was inhibited by DPI (Fig. 5). Thus, activation of both PERK and IRE1 pathways by PMA is dependent on NADPH oxidase ROS production and not on PKC activation *per se*.

**Fig 6:**
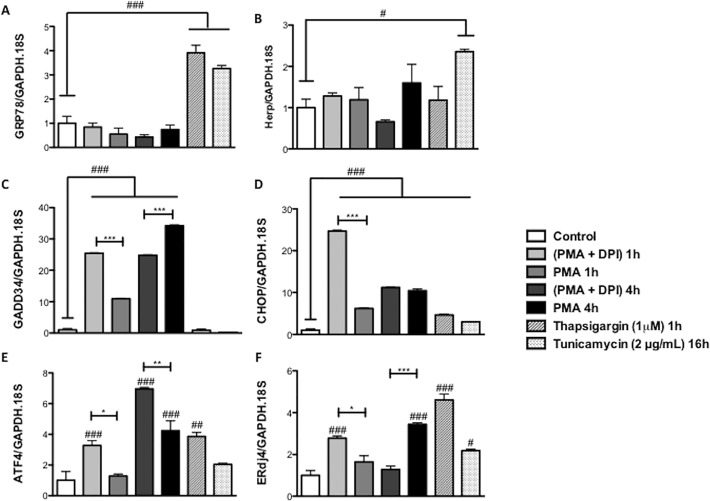
Effect of ROS production on the expression of genes involved in the unfolded protein response (UPR). GAPDH and 18S were used as reference control genes. Results are from 6 independent experiments. Thapsigargin (1μM) 1h and Tunicamycin (2 μg/ml) 16h were used as positive controls. (***) Indicates p <0.001; (**) Indicates p <0.01 and (*) Indicates p <0.05; (###) indicates p<0.001 *vs* control group; (##) indicates p<0.01 *vs* control group; (#) indicates p<0.05 *vs* control group.

Finally, we examined by real time PCR if the expression of genes regulated by the UPR (GRP78, Herp, GADD34, CHOP, ATF4 and ERdj4) is altered by PMA in dHL60 cells. Expression of GRP78 and Herp increased when dHL60 cell were treated with known ER stress-inducing compounds tunicamycin and/or thapsigargin, but PMA treatment had no significant effect (Fig. 6A,B). GADD34 expression was increased in dHL60 cells stimulated with PMA for 1 and 4h. DPI enhanced this effect at 1h, but partially inhibited this increase at the 4h time point (Fig. 6C). CHOP expression was also increased by PMA and similar to GADD34 DPI enhanced this effect at the 1h time point (Fig. 6D). The expression of ATF4 was increased by PMA at 4h and DPI potentiated the PMA effect at both 1 h and 4 h (Fig. 6E). In contrast, DPI inhibited PMA induction of the co-chaperone protein ERdj4 at 4h of treatment (Fig. 6F).

## Discussion

It was once thought that neutrophils were terminally differentiated cells lacking transcription activity and protein synthesis. More recently, however, it has been reported that neutrophils in fact exhibit a high capacity to carry out *de novo* synthesis of various proteins, such as cytokines and chemokines with immunomodulatory properties [1; 48]. The neutrophil ER is functional and should be considered as a key organelle in mediating cell viability, especially in the activated state, when the production of secretory proteins increases [49]. However, activated neutrophils are also large producers of ROS primarily via NADPH oxidase. Given that ROS can induce ER stress [14; 15; 30–32], a situation that leads to an increase unfolded and misfolded proteins in the organelle, we sought to determine if ER stress occurs in neutrophils exposed to chronic hyperglycemia or when acutely activated.

Chronic hyperglycemia is known to induce ROS production in many cell types, but failed to increase ROS levels or to cause ER stress in dHL60 cells. This is likely due to glucose transporter (GLUT) expression in these cells. Neutrophils and dHL60 express GLUT 1 exclusively [43; 50–52], which has a low Km and gets saturated such that glucose transport does not increase despite high extracellular levels, protecting the cell against glucotoxicity. In contrast, GLUT 2 has a high Km for glucose (~17 mM), so the higher the concentration of glucose in the extracellular media, the higher the transport into the cell [52]. Pancreatic β-cells that express GLUT2 respond to high glucose concentration by producing and secreting more insulin. Thus, hyperglycemia by stimulating insulin biosynthesis and ROS production causes ER stress in these cells [39; 52; 53].

We found that dHL60 cells treated with the PKC activator, PMA, showed large increases in ROS production that correlated with induction of ER stress markers. It has been shown previously that fMLP triggers ROS production in dHL60 which is accompanied by calcium influx [54]. It is known that ER stress can be caused by reducing ER calcium levels such as with the compound thapsigargin that inhibits the ER Ca^2^-ATPase [55–57], or by ionomycin a calcium ionophore that increases the influx of Ca^2+^ [58]. However, PMA failed to induce calcium influx and, thus its ability to induce ER stress in dHL60 occurred independently of calcium changes. Importantly, DPI a specific inhibitor of NADPH oxidase protein complex assembly, which does not inhibit PKC [48], prevented ROS induction and activation of the ER stress.

In response to ER stress, three UPR signaling pathways are activated that are initiated by PERK, IRE1 and ATF6 proteins [59]. We monitored two of these pathways in response to PMA treatment. The PERK pathway was monitored by the levels of eiF2α phosphorylation. Phosphorylation of eiF2α results in a transient reduced general protein synthesis that is cytoprotective since this will limit the amount of new protein synthesis in the ER. PMA induced a rapid (within 1h) and large increase in phospho-eIF2 levels that was completely inhibited by DPI and is thus NADPH oxidase dependent. Reduced protein synthesis caused by eiF2α phosphorylation would be expected to result in increased ATF4 and GADD34 protein levels [60]. However, no changes in these proteins were observed after 1 h or 4h PMA treatment. The reason for this is unclear, although the inhibition of protein synthesis may not be sufficient to allow for enhanced ATF4 translation and the effect on general protein translation should be measured in future experiments. Interestingly, ATF4, GADD34 and CHOP mRNA levels were increased by PMA treatment, although this was not inhibited by DPI, suggesting an NADPH oxidase (and ER stress) independent effect. Indeed, in some cases DPI potentiated the effect of PMA, which might relate to other cellular effects of DPI, such as inhibition of O_2_^o^and H_2_O_2_ production by mitochondria [61], induction of DNA damage [62] and increase in mRNA and protein levels related to cell death [63].

Phosphorylation of eiF2α can be mediated by kinases other than PERK (10) and therefore can occur independent of ER stress. That PMA does indeed induce ER stress is apparent from the large induction of sXBP1 mRNA and GRP78 protein levels, two bona fide markers of ER stress (10). The fact that both changes are completely inhibited by DPI indicates that the effect is dependent on NADPH oxidase activation and likely ROS production. In the second pathway investigated, IRE1 splices cytosolic XBP1 mRNA that results in production of the XBP1 transcription factor. XBP1 regulates numerous genes including ER chaperones, genes involved in ERAD, genes involved in ER expansion and secretory protein trafficking [64–71]. Interestingly however, neither GRP78 nor the ERAD gene Herp were affected by PMA treatment for either 1 or 4 h, despite a clear increase in sXBP1 mRNA levels. This is surprising since XBP1 is known to contribute to regulating the induction of these genes in response to ER stress and GRP78 protein levels were increased. It is possible that the changes in mRNA levels for these genes occur at some point during the 1h PMA treatment and levels are reduced to basal subsequently. The positive control treatments (tunicamycin and thapsigargin) increased the levels of these genes, thus the lack of effect is not due to technical issues with the assay. However, PMA did induce another gene regulated by the IRE1α/XBP1 pathway, the co-chaperone ERdj4 that has multiple functions in the ER lumen, such as removal of newly synthesized unfolded and/or misfolded proteins by promoting the GRP78 ATPase activity [72, 73] or by enhancing the activity of the ERAD machinery [74, 75]. At 4h of PMA treatment the induction of ERdj4 expression in response to PMA was completely inhibited, indicating the effect was due to NADPH oxidase activation.

In summary, we have shown that neutrophil-like HL60 cells undergo ER stress when the NADPH oxidase is activated. Thus, in response to activation dHL60 cells are eliciting an adaptive system (the UPR) to maintain ER homeostasis and cell survival during the potentially detrimental presence of excess ROS production. We suggest that the UPR is vital for neutrophils to perform their function in the immune response. The UPR will allow cells to maintain survival for potentially multiple encounters with pathogens and likely more importantly, to maintain the ability to synthesize and secrete cytokines and chemokines as part of the inflammatory response. Future studies should investigate the UPR in the context of activation in primary neutrophils. Knowledge of UPR activation may lead to a better understanding of neutrophil functions, such as phagocytosis, cytokine and chemokine production and cell survival

## Supporting Information

S1 Fig Effect of Hyperglycemia and PMA on eIF2α phosphorylation (n = 3). Membranes were cut where indicated for primary antibody incubations.
(TIF)

S2 Fig Effect of Hyperglycemia and PMA on the splicing of XPB1. (n = 3) (TIF)

S3 Fig Effect of ROS production on the phosphorylation of eIF2α (n = 3). Membranes were cut where indicated for primary antibody incubations.
(TIF)

S4 Fig Ponceau staining of the membranes (n = 6). Membranes were cut where indicated for primary antibody incubations.
(TIF)

S5 Fig Effect of ROS production on the levels of GRP78 and GADD34 (n = 6). Membranes were cut as indicated in the ponceau staining (S4 Fig.) for primary antibody incubations.
(TIF)

S6 Fig Effect of ROS production on the levels of ATF4. Υ Tubulin was used as a loading control (n = 6). Membranes were cut as indicated in the ponceau staining (S4 Fig.) for primary antibody incubations.
(TIF)

S7 Fig Effect of ROS production on the splicing of XBP1 (n = 6). GAPDH was used as a reference control gene (n = 3).
(TIF)

S8 Fig Graphs representing qPCR amplification and melting curves of the genes involved in the UPR (GRP78, GADD34, CHOP, ATF4, ERdj4 and Herp) and two reference control genes (GAPDH and 18S). (ZIP)

S1 Table Median of fluorescence intensity (DHR 123: λ excitation: 488nm; λ emission 520nnm – Flow cytometer analysis). (PDF)

S2 Table Intracellular calcium measurements (Indo 1: λ excitation = 331nm; λ emission = 410 nm – Fluorimeter analysis). (PDF)

S3 Table ER calcium measurements (Indo 1: λ excitation = 331nm; λ emission = 410 nm Fluorimeter analysis). (PDF)

S4 Table The cycle threshold (Ct) mean values of the UPR (GRP78, GADD34, CHOP, ATF4, ERdj4 and Herp) and reference control (GAPDH and 18S) genes. (PDF)
