# Supplementary figures and images for: NADPH Oxidase-Dependent Production of Reactive Oxygen Species Induces Endoplasmatic Reticulum Stress in Neutrophil-Like HL60 Cells
Source: PLoS One. 2015 Feb 10;10(2):e0116410. doi: 10.1371/journal.pone.0116410 (PMC4323339; doi:10.1371/journal.pone.0116410)

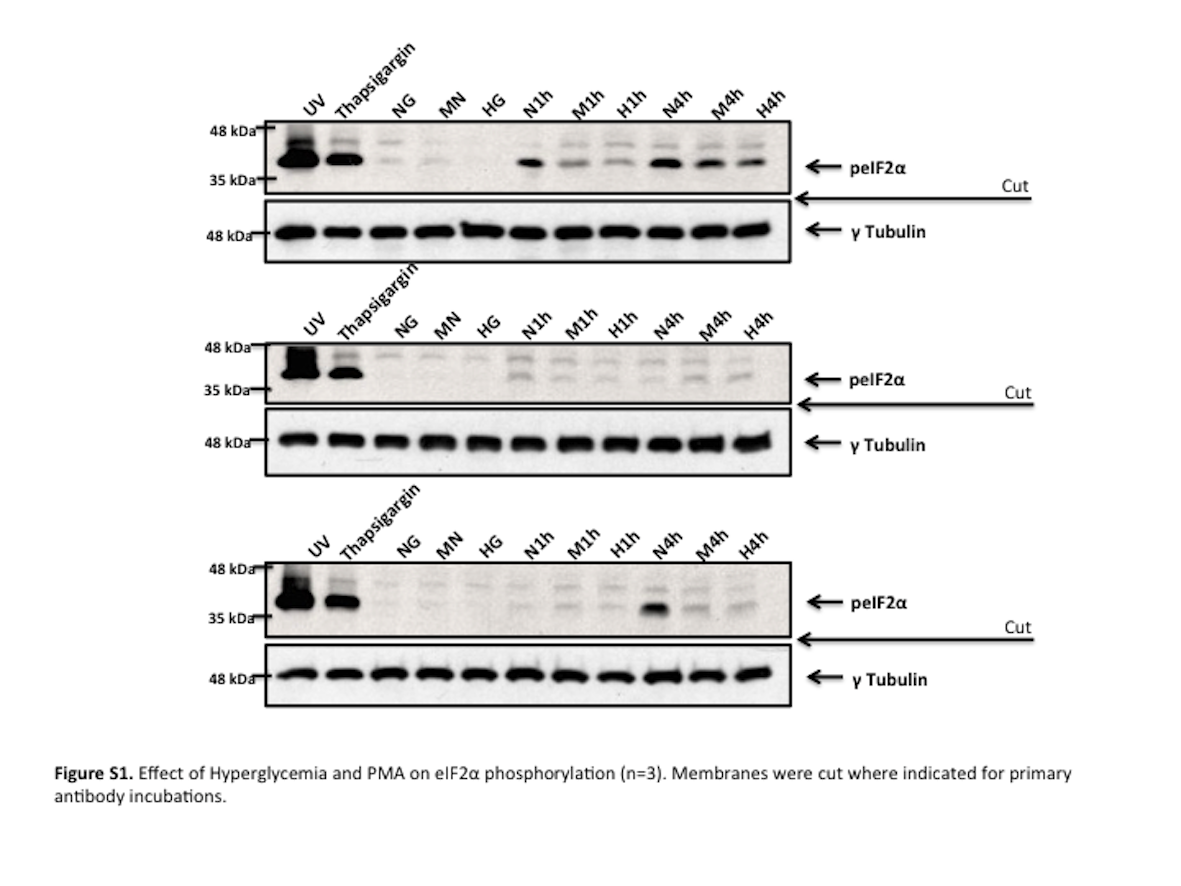

Supplement: S1 Fig — Membranes were cut where indicated for primary antibody incubations. (TIF) [file pone.0116410.s001.tif]

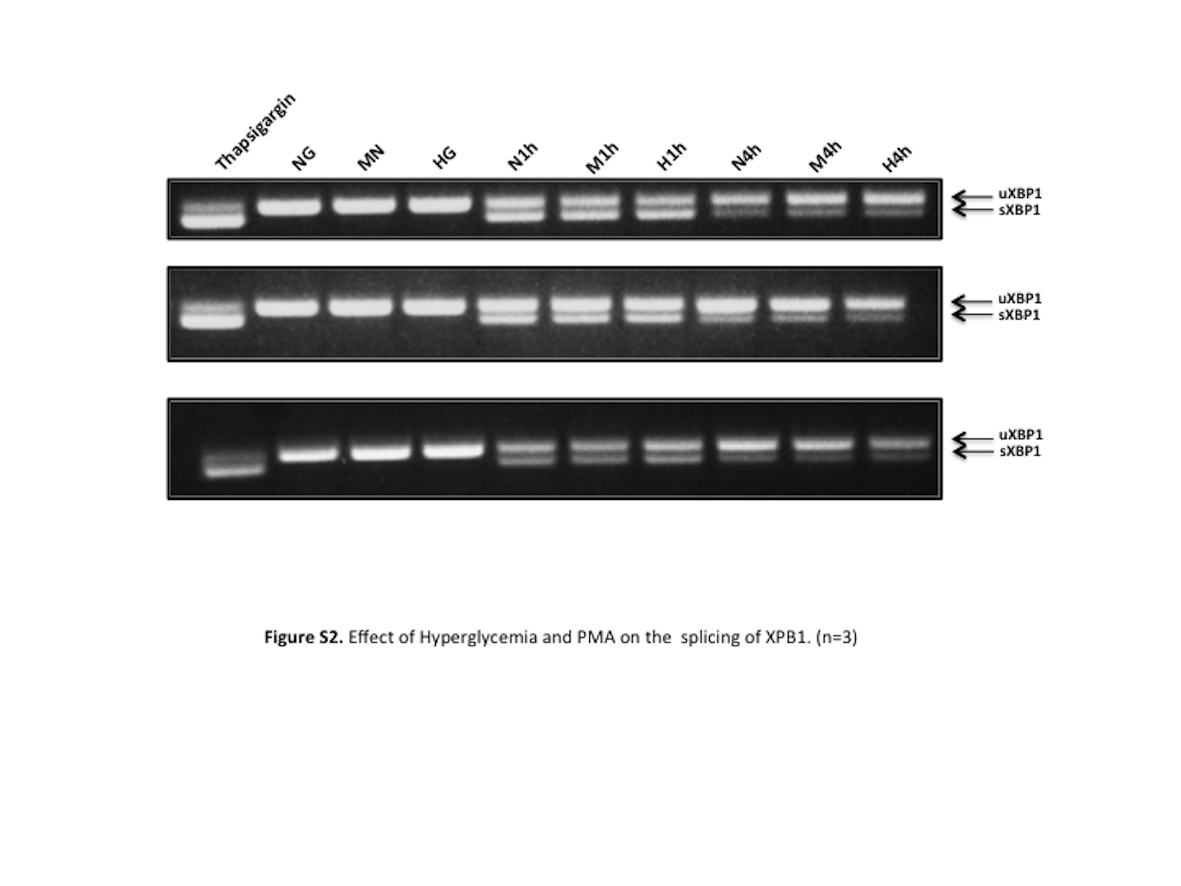

Supplement: S2 Fig — (TIF) [file pone.0116410.s002.tif]

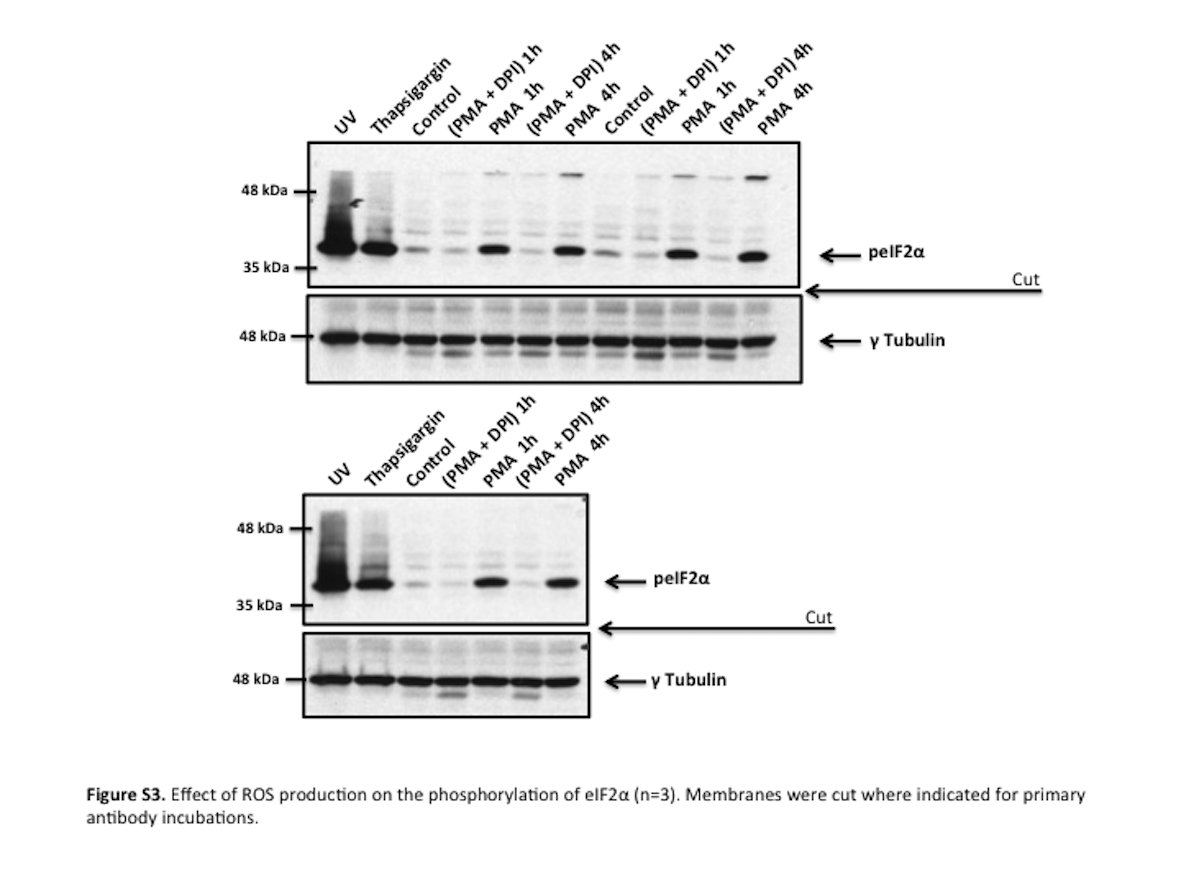

Supplement: S3 Fig — Membranes were cut where indicated for primary antibody incubations. (TIF) [file pone.0116410.s003.tif]

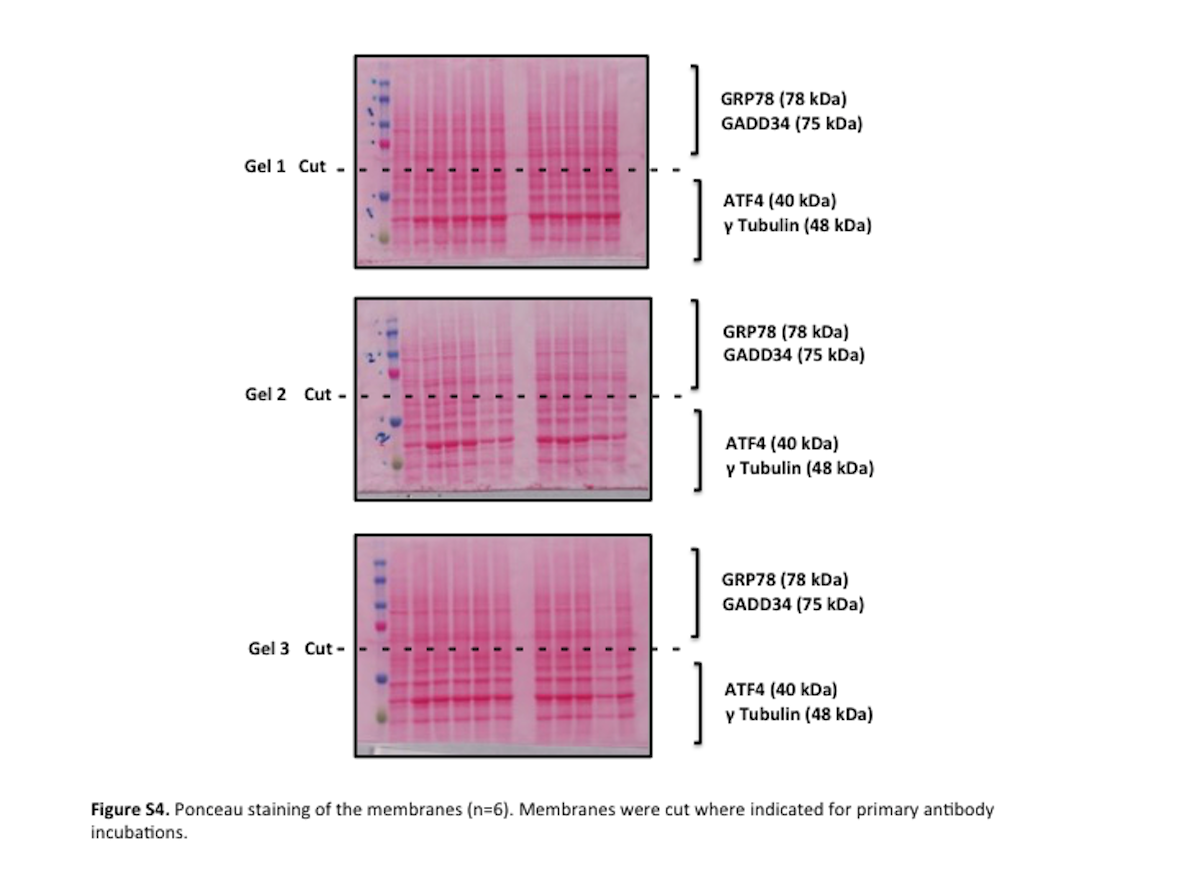

Supplement: S4 Fig — Membranes were cut where indicated for primary antibody incubations. (TIF) [file pone.0116410.s004.tif]

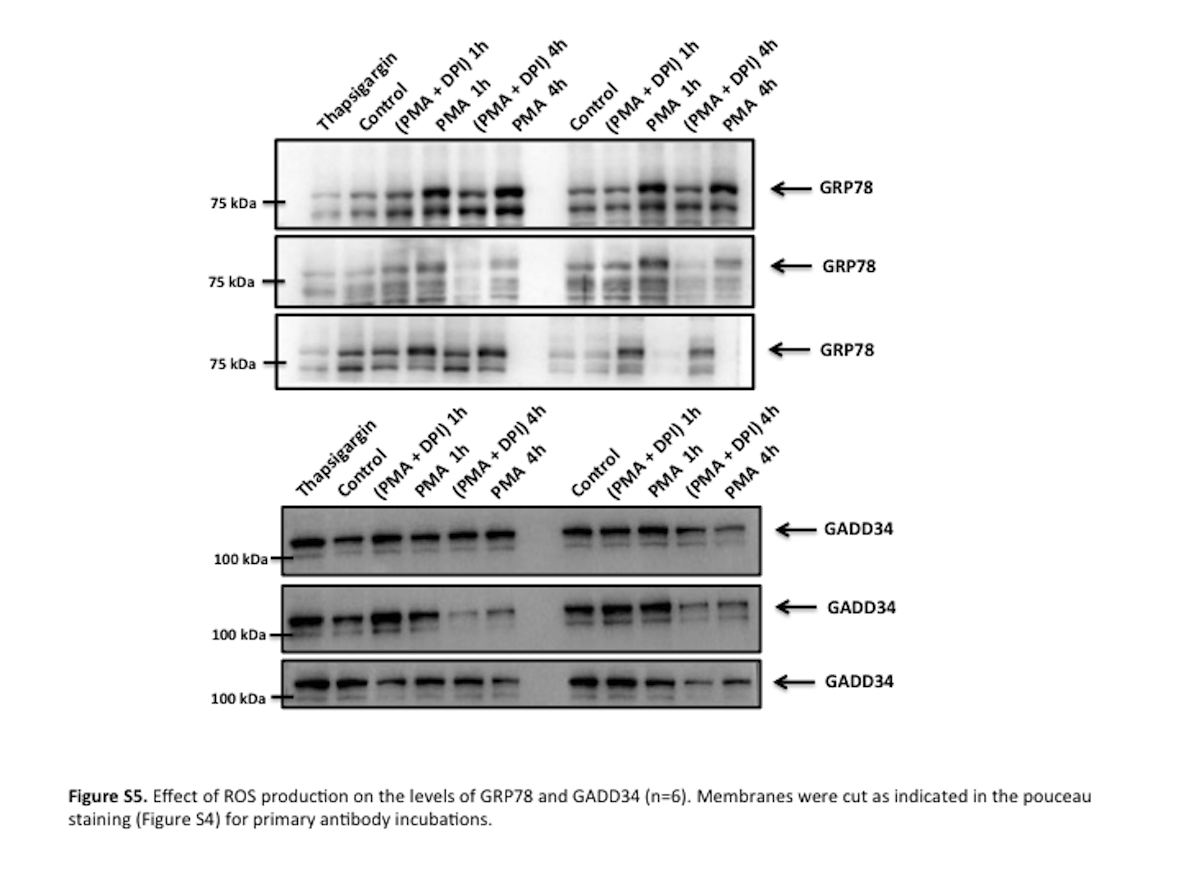

Supplement: S5 Fig — Membranes were cut as indicated in the ponceau staining (S4 Fig.) for primary antibody incubations. (TIF) [file pone.0116410.s005.tif]

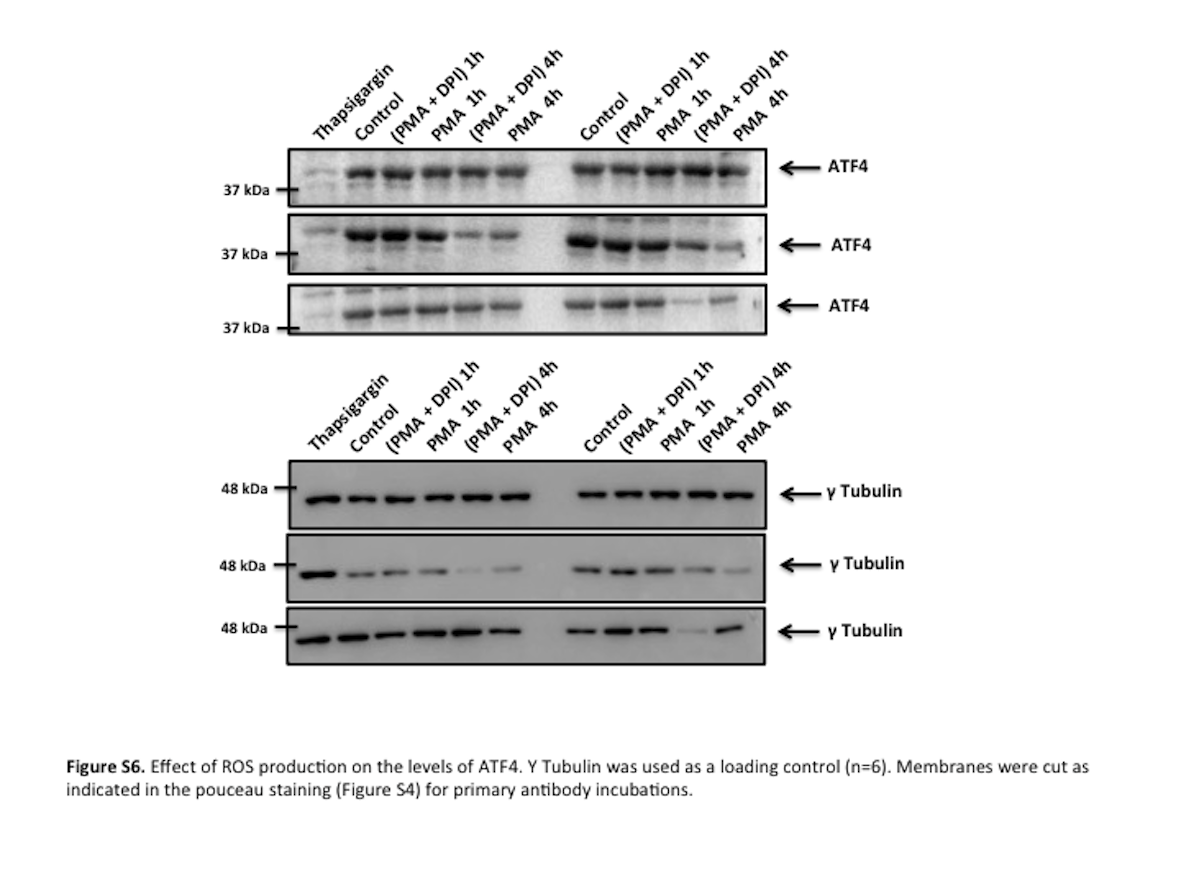

Supplement: S6 Fig — Υ Tubulin was used as a loading control (n = 6). Membranes were cut as indicated in the ponceau staining (S4 Fig.) for primary antibody incubations. (TIF) [file pone.0116410.s006.tif]

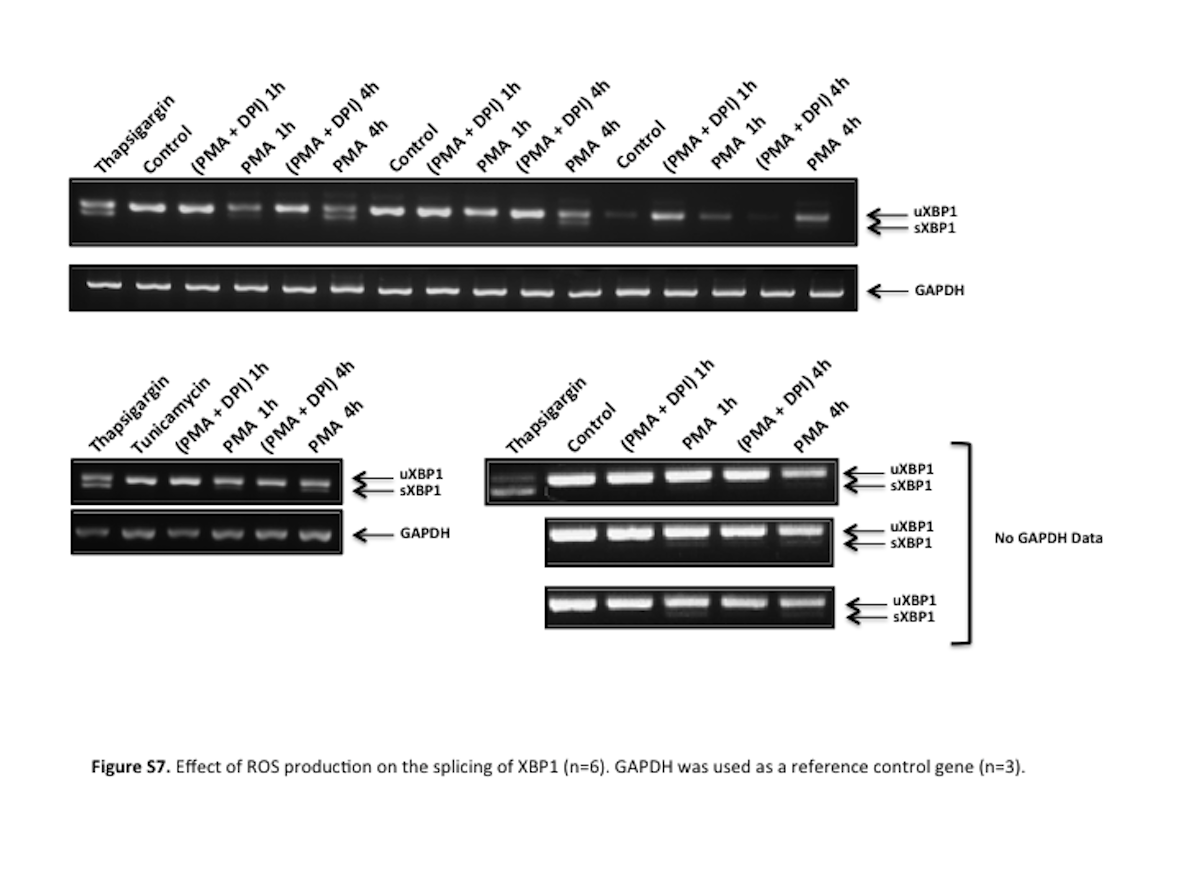

Supplement: S7 Fig — GAPDH was used as a reference control gene (n = 3). (TIF) [file pone.0116410.s007.tif]

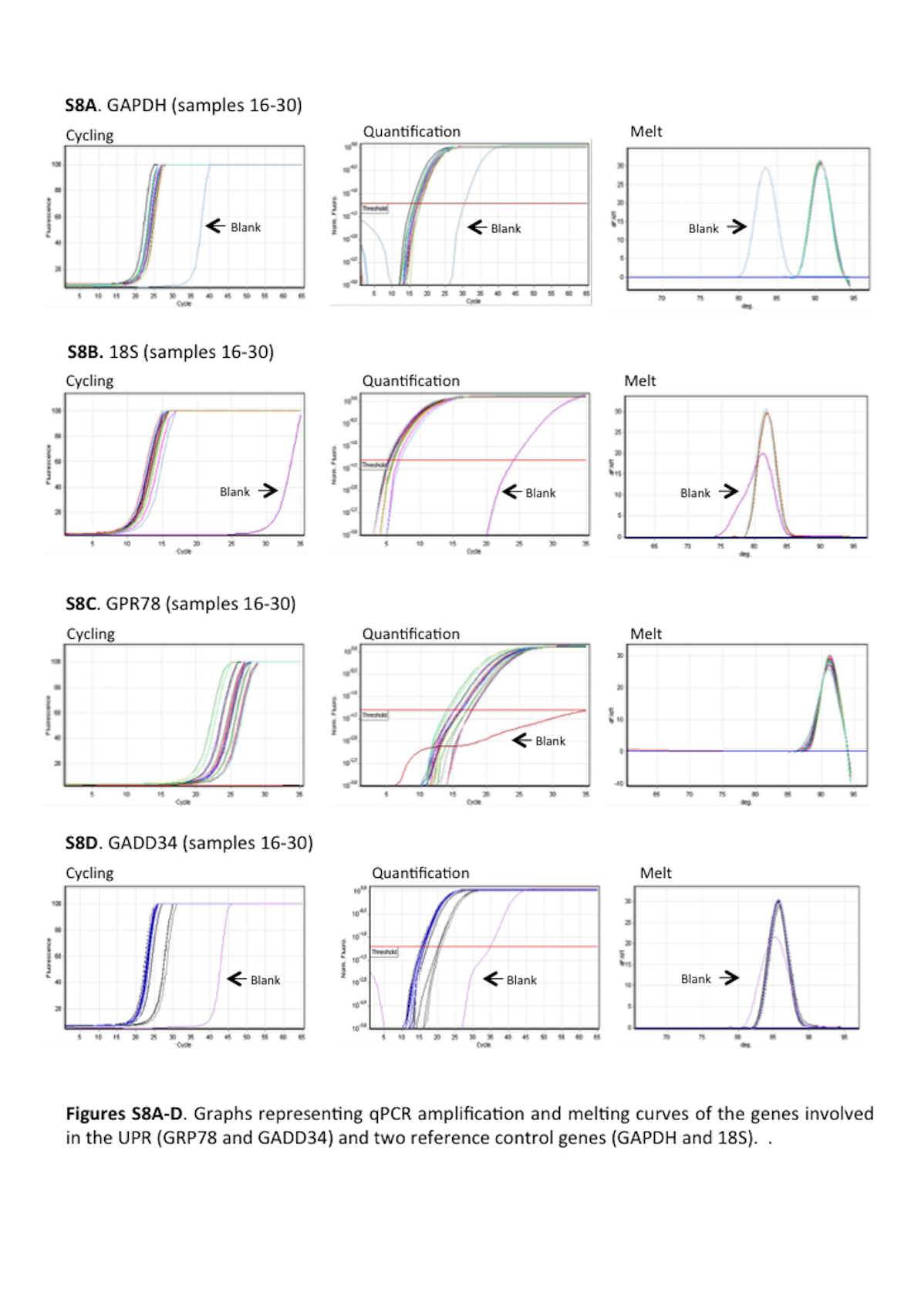

Supplement: S8 Fig — (ZIP) [file pone.0116410.s008.zip › Figure S8A-D.tif]

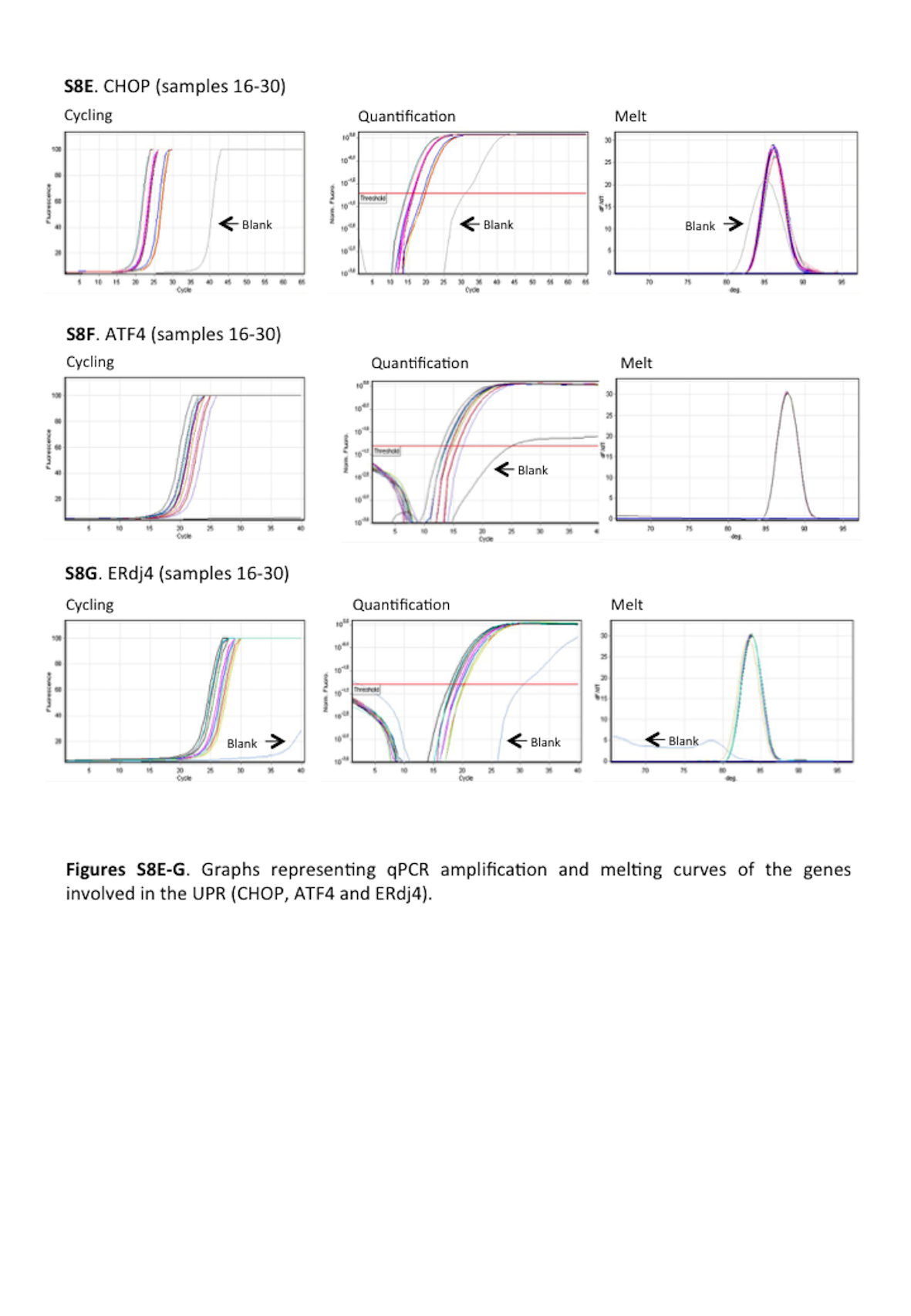

Supplement: S8 Fig — (ZIP) [file pone.0116410.s008.zip › Figure S8E-G.tif]

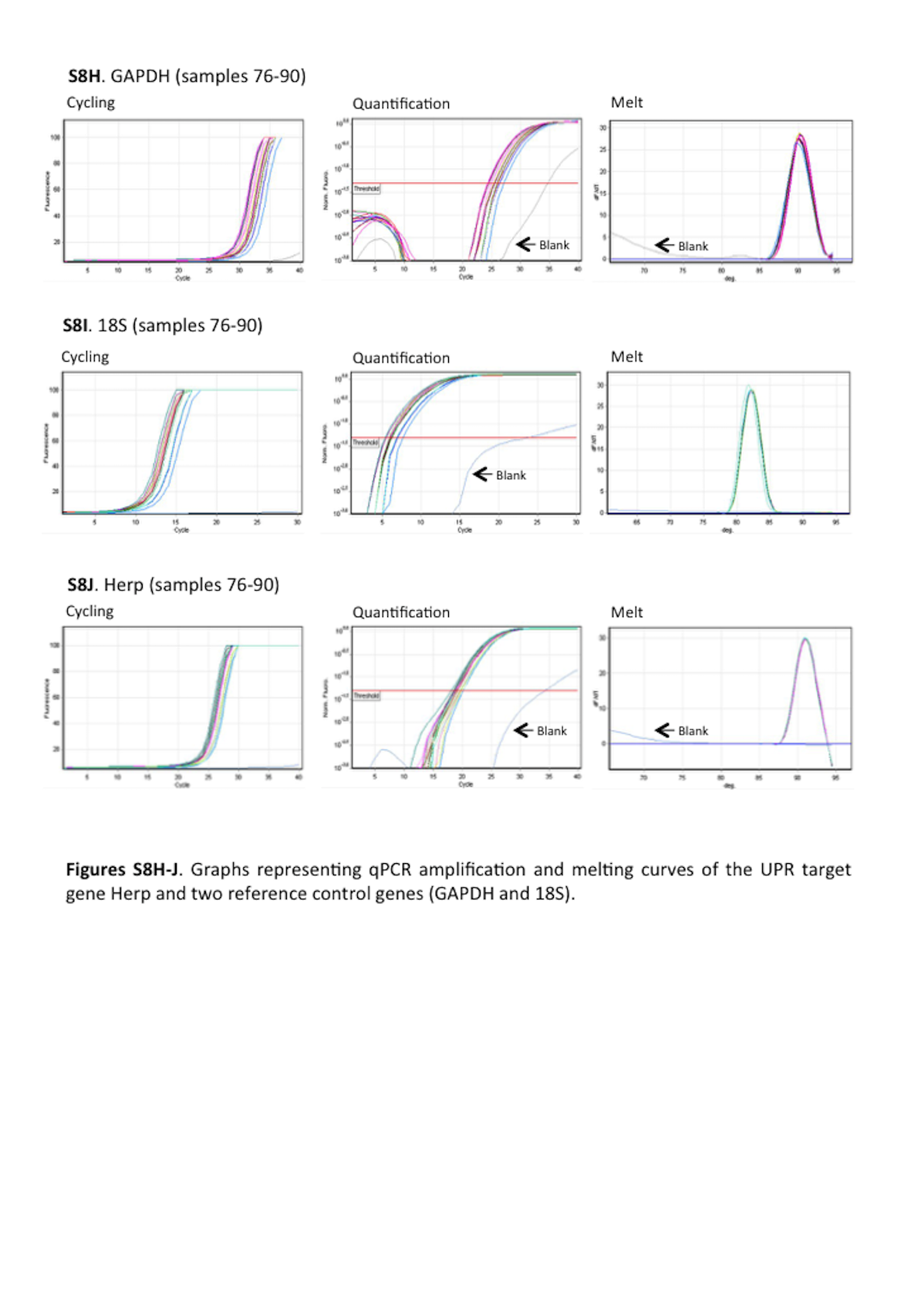

Supplement: S8 Fig — (ZIP) [file pone.0116410.s008.zip › Figure S8H-J.tif]
