# Supplementary material for: NADPH Oxidase-Dependent Production of Reactive Oxygen Species Induces Endoplasmatic Reticulum Stress in Neutrophil-Like HL60 Cells
Source: PLoS One. 2015 Feb 10;10(2):e0116410. doi: 10.1371/journal.pone.0116410 (PMC4323339; doi:10.1371/journal.pone.0116410)
Supplement: S1 Table — (PDF) [file pone.0116410.s009.pdf]

Table S1. Median of fluorescence intensity (DHR 123: λ excitation: 488nm; λ emission 520nnm – Flow cytometer analysis).

	HL60	HL60 PMA +DPI	HL60 PMA	dHL60 NG	dHL60 NG PMA + DPI	dHL60 NG + PMA
**N=1**	11170	10926	11975	2455	3317	18168
**N=2**	6273	10058	10540	3462	5195	7312
**N=3**	11392	11773	10414	2935	5334	17637
**N=4**	7902	11702	13063	1915	3904	11201
**N=5**	11479	13827	13884	3507	8542	17297
**N=6**	6277	10076	8067	2767	5516	14267
**Average**	**9082,166667**	**11393,66667**	**11323,83333**	**2840,166667**	**5301,333333**	**14313,66667**
**STDEV**	**2553,129798**	**1407,056313**	**2100,110799**	**608,2724444**	**1813,863023**	**4324,694563**
**ERROR**	**1042,310875**	**574,4283342**	**857,3666433**	**248,3261856**	**740,5064783**	**1765,549162**

	dHL60 Mannitol	dHL60 Mannitol + PMA + DPI	dHL60 Mannitol + PMA	dHL60 HG	dHL60 HG + PMA + DPI	dHL60 HG + PMA
**N=1**	2216	4152	13756	2434	3822	14753
**N=2**	4163	5750	7665	3547	4730	8410
**N=3**	2947	6410	19114	3335	6352	18197
**N=4**	1756	4115	12546	1661	3356	11274
**N=5**	3521	8641	16105	4033	6892	15153
**N=6**	2611	6498	16811	2913	6641	15519
**Average**	**2869**	**5927,666667**	**14332,83333**	**2987,166667**	**5298,833333**	**13884,33333**
**STDEV**	**876,0554777**	**1696,491753**	**4003,172513**	**848,3372953**	**1531,53262**	**3476,13266**
**ERROR**	**357,6481511**	**692,5898578**	**1634,288335**	**346,3322506**	**625,245574**	**1419,125216**
