# Supplementary material for: NADPH Oxidase-Dependent Production of Reactive Oxygen Species Induces Endoplasmatic Reticulum Stress in Neutrophil-Like HL60 Cells
Source: PLoS One. 2015 Feb 10;10(2):e0116410. doi: 10.1371/journal.pone.0116410 (PMC4323339; doi:10.1371/journal.pone.0116410)
Supplement: S2 Table — (PDF) [file pone.0116410.s010.pdf]

Table S2. Intracellular calcium measurements (Indo 1: λ excitation= 331nm; λ emission= 410 nm – Fluorimeter analysis).

Intracellular						
**Sample**	**F (Basal)**	**F (min)**	**F (Peak)**	**F (max)**	**[Ca^{2+}] Basal**	**[Ca^{2+}] Peak**
**dHL60 NG (n=1)**	895,2	187,125	951,7	1030	1,313195475	2,441171775
**dHL60 NG (n=2)**	1079	211,075	1229	1313	0,927270299	3,02953869
**dHL60 NG (n=3)**	1063	225,2166667	1184	1387	0,646437757	1,180767652
**dHL60 NG (n=4)**	1052	228,1083333	1201	1392	0,605802696	1,273418412
**dHL60 MN (n=1)**	1065	215,5	1172	1277	1,001768868	2,277380952
**dHL60 MN (n=2)**	1390	254,9333333	1514	1561	1,659454191	6,697163121
**dHL60 MN (n=3)**	1060	244,2833333	1208	1464	0,504775165	0,941129557
**dHL60 MN (n=4)**	1077	243,15	1217	1457	0,548585526	1,014427083
**dHL60 HG (n=1)**	1073	223,2416667	1246	1338	0,801658805	2,779234601
**dHL60 HG (n=2)**	1118	231,45	1241	1387	0,823931227	1,728681507
**dHL60 HG (n=3)**	1048	234,7833333	1181	1592	0,373720895	0,575557583
**dHL60 HG (n=4)**	1066	237,6833333	1164	1475	0,506306031	0,744627546

**Basal**	**dHL60 NG**	**dHL60 MN**	**dHL60 HG**
	1,313195475	1,001768868	0,801658805
	0,927270299	1,659454191	0,823931227
	0,646437757	0,504775165	0,373720895
	0,605802696	0,548585526	0,506306031
**Average**	0,873176557	0,928645938	0,626404239
**STDEV**	0,326313778	0,53651344	0,222113729
**Error**	0,163156889	0,26825672	0,111056865

**Peak (fMLP)**	**dHL60 NG**	**dHL60 MN**	**dHL60 HG**
	2,441171775	2,277380952	2,779234601
	3,02953869	6,697163121	1,728681507
	1,180767652	0,941129557	0,575557583
	1,273418412	1,014427083	0,744627546
**Average**	1,981224132	2,732525178	1,457025309
**STDEV**	0,904108172	2,713329419	1,017600188
**Error**	0,452054086	1,35666471	0,508800094

[Ca^{2+}] intracellular was calculated using the following formula as previously reported in MacDougall et al., 1988 [33]:

$$[Ca^{2+}]_i = 250 \text{ nM } (F - Fmin)/(Fmax - F)*0{,}001$$
