# Supplementary material for: NADPH Oxidase-Dependent Production of Reactive Oxygen Species Induces Endoplasmatic Reticulum Stress in Neutrophil-Like HL60 Cells
Source: PLoS One. 2015 Feb 10;10(2):e0116410. doi: 10.1371/journal.pone.0116410 (PMC4323339; doi:10.1371/journal.pone.0116410)
Supplement: S3 Table — (PDF) [file pone.0116410.s011.pdf]

Table S3. ER calcium measurements (Indo 1: λ excitation= 331nm; λ emission= 410 nm – Fluorimeter analysis).

ER				
**Sample**	**F (min)**	**F (Peak)**	**F (max)**	**$[Ca^{2+}]$ ER**
**dHL60 NG (n=1)**	288,8916667	655,4	1347	0,132485661
**dHL60 NG (n=2)**	215,3	748,1	1268	0,256203116
**dHL60 NG (n=3)**	198,6691667	707,5	1287	0,21951287
**dHL60 NG (n=4)**	218,9583333	787,5	1357	0,249579309
**dHL60 NG (n=5)**	153,7358333	579,7	1014	0,24520157
**dHL60 MN (n=1)**	267	889	1642	0,206507304
**dHL60 MN (n=2)**	255,3083333	933,3	1571	0,265795698
**dHL60 MN (n=3)**	209,35	807,8	1366	0,268026693
**dHL60 MN (n=4)**	208,0433333	897,8	1525	0,274934896
**dHL60 MN (n=5)**	212,1916667	718,6	1399	0,186070081
**dHL60 HG (n=1)**	285,0416667	865,2	1809	0,153676185
**dHL60 HG (n=2)**	209,1916667	782	1308	0,272247307
**dHL60 HG (n=3)**	199,5666667	715,8	1209	0,261675453
**dHL60 HG (n=4)**	203,3258333	783	1419	0,227859342
**dHL60 HG (n=5)**	164,8608333	585,1	1098	0,204834844

**ER**	**dHL60 NG**	**dHL60 MN**	**dHL60 HG**
	0,132485661	0,206507304	0,153676185
	0,256203116	0,265795698	0,272247307
	0,21951287	0,268026693	0,261675453
	0,249579309	0,274934896	0,227859342
	0,24520157	0,186070081	0,204834844
**Average**	0,220596505	0,240266934	0,224058626
**STDEV**	0,0511809	0,040930432	0,047631035
**Error**	0,022888794	0,018304646	0,021301246

ER $[Ca^{2+}]$ was calculated using the following formula as previously reported in MacDougall et al., 1988 [33]:

$$[Ca^{2+}]_{ER} = 250\ nM\ (F - Fmin)/(Fmax - F)*0,001$$
