# Supplementary material for: NADPH Oxidase-Dependent Production of Reactive Oxygen Species Induces Endoplasmatic Reticulum Stress in Neutrophil-Like HL60 Cells
Source: PLoS One. 2015 Feb 10;10(2):e0116410. doi: 10.1371/journal.pone.0116410 (PMC4323339; doi:10.1371/journal.pone.0116410)
Supplement: S4 Table — (PDF) [file pone.0116410.s012.pdf]

Table S4. The cycle threshold (Ct) mean values of the UPR (GRP78, GADD34, CHOP, ATF4, ERdj4 and Herp) and reference control (GAPDH and 18S) genes.

Sample	GAPDH	18S	GRP78	GADD34	CHOP	ATF4	ERdj4
**16**	16.69	5.37	16.22	21.03	19.78	16.56	20.24
**17**	17.92	6.10	16.26	20.51	19.55	15.55	20.44
**18**	16.46	5.29	15.87	20.23	19.01	15.53	19.57
**19**	17.28	5.37	17.06	17.13	16.35	15.63	18.71
**20**	16.55	5.12	15.94	16.24	16.27	14.75	19.31
**21**	17.18	4.37	16.21	16.30	16.08	14.45	18.32
**22**	17.35	5.08	16.06	15.83	14.54	13.83	18.37
**23**	17.41	5.12	16.32	15.71	14.84	14.40	18.40
**24**	16.60	5.86	16.17	15.66	14.52	13.72	18.50
**25**	17.15	6.65	17.75	16.20	16.29	14.42	19.27
**26**	15.53	5.34	15.99	15.29	16.16	14.40	17.78
**27**	16.30	5.93	16.27	15.35	16.10	12.94	18.22
**28**	18.10	5.91	17.90	16.82	16.56	13.56	20.24
**29**	17.86	6.05	17.83	16.33	16.50	13.66	20.13
**30**	16.78	5.24	17.13	15.58	15.68	12.88	19.66
**Blank**	30.47	24.15	ND	34.94	31.17	25.11	ND

Sample	GAPDH	18S	Herp
**76**	26.05	6.23	19.70
**77**	25.35	5.93	19.17
**78**	26.35	6.99	19.82
**79**	24.36	5.50	18.73
**80**	24.76	5.91	18.81
**81**	27.09	7.65	20.13
**82**	24.51	5.32	18.09
**83**	25.03	5.97	18.72
**84**	25.97	6.22	18.93
**85**	24.28	5.98	18.90
**86**	25.02	6.10	18.49
**87**	25.51	6.89	18.63
**88**	25.50	5.92	19.66
**89**	24.46	5.86	19.64
**90**	26.45	6.30	20.12
**Blank**	34.67	23.93	34.27
